# Recent Development of Rhenium-Based Materials in the Application of Diagnosis and Tumor Therapy

**DOI:** 10.3390/molecules28062733

**Published:** 2023-03-17

**Authors:** Qingwen Qi, Qian Wang, Yuhao Li, Dionisio Zaldivar Silva, Maria Eliana Lanio Ruiz, Ruizhuo Ouyang, Baolin Liu, Yuqing Miao

**Affiliations:** 1 School of Materials and Chemistry, Institute of Bismuth and Rhenium, University of Shanghai for Science and Technology, Shanghai 200093, China; qqw136386303@163.com (Q.Q.); wangqian980314@163.com (Q.W.); ouyangrz@usst.edu.cn (R.O.); 2 USST-UH International Joint Laboratory for Tumor Diagnosis and Energy Treatment, University of Shanghai for Science and Technology, Shanghai 200093, China; mlanio@fbio.uh.cu (M.E.L.R.); blliuk@163.com (B.L.); 3 Faculty of Biology, University of Havana, Havana 10400, Cuba; 4 School of Health Science and Engineering, University of Shanghai for Science and Technology, Shanghai 200093, China

**Keywords:** rhenium, complexes, nanomaterials, cancer therapy, imaging

## Abstract

Rhenium (Re) is widely used in the diagnosis and treatment of cancer due to its unique physical and chemical properties. Re has more valence electrons in its outer shell, allowing it to exist in a variety of oxidation states and to form different geometric configurations with many different ligands. The luminescence properties, lipophilicity, and cytotoxicity of complexes can be adjusted by changing the ligand of Re. This article mainly reviews the development of radionuclide ^188^Re in radiotherapy and some innovative applications of Re as well as the different therapeutic approaches and imaging techniques used in cancer therapy. In addition, the current application and future challenges and opportunities of Re are also discussed.

## 1. Introduction

With the rapid development of modern science and technology, people’s living conditions have been greatly improved in all aspects, so the life span of human beings has been significantly extended. Many studies have shown that the incidence of cancer often increases with age [1,2,3], which may be due to long-term exposure to carcinogens [4], in vivo environments being more conducive to malignant cell proliferation [5], cumulative mutation [6], etc. In any case, cancer has gradually become one of the most threatening and deadly diseases to human health. Therefore, researchers have invested a lot of energy and material resources into the development of cancer treatment, and have made some achievements. In the diagnosis of cancer, several imaging techniques [7] such as computed tomography (CT) [8,9], magnetic resonance imaging (MRI) [10], fluorescence imaging [11,12,13,14], photoacoustic (PA) imaging [15], infrared thermal imaging [16], ultrasound (US) imaging [17,18,19], positron emission tomography (PET) [20,21,22], and single photon emission computed tomography (SPECT) [23,24] have been developed. Cancer detection has become more sensitive and accurate. In the treatment of cancer, radiotherapy (RT) [25,26], chemotherapy [27,28,29,30], photodynamic therapy (PDT) [31,32,33,34,35,36], photothermal therapy (PTT) [37,38,39], sonodynamic therapy (SDT) [40,41,42], immunotherapy [43,44,45], starvation therapy (ST) [33,46,47], magnetothermal therapy (MHT) [48,49], and other therapies have also shown good anticancer effects. As a remarkable diagnostics reagent candidate, the Re element has attracted the attention of many researchers because of its unique physical and chemical properties.

**Figure 1:**
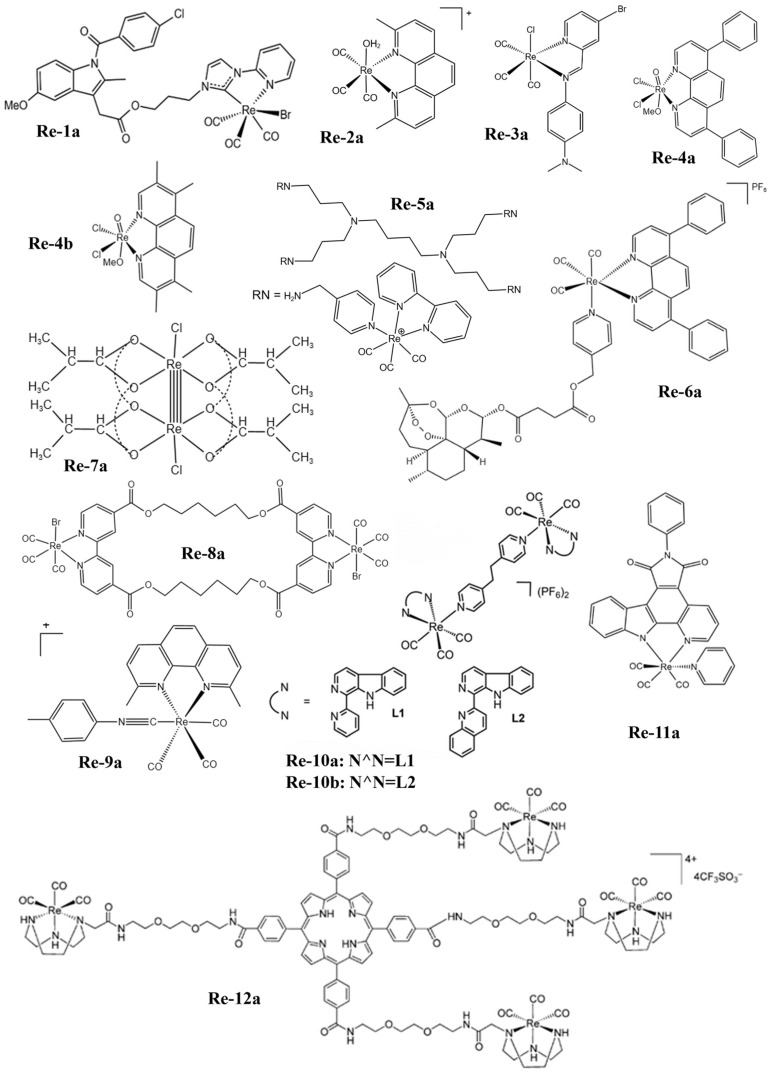
The structures of some Re complexes (Re-1a−Re-12a) involved in this review.

**Figure 2:**
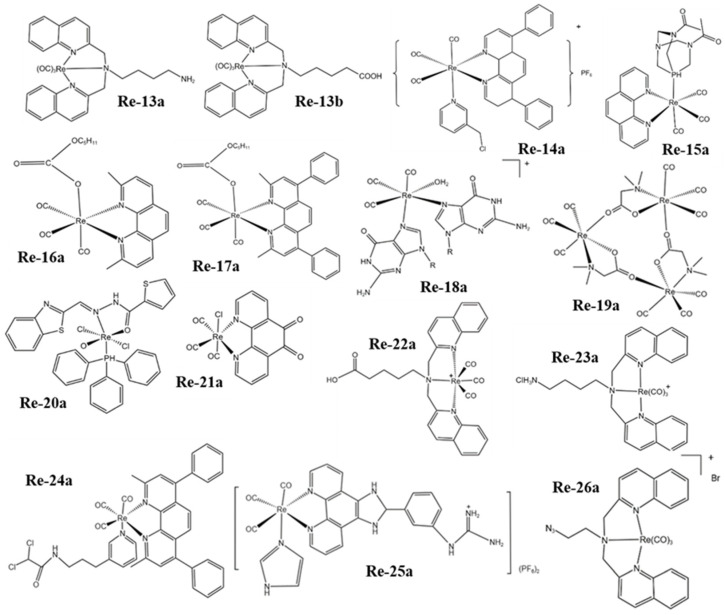
The structures of some Re complexes (Re-13a−Re-26a) involved in this review.

**Figure 3:**
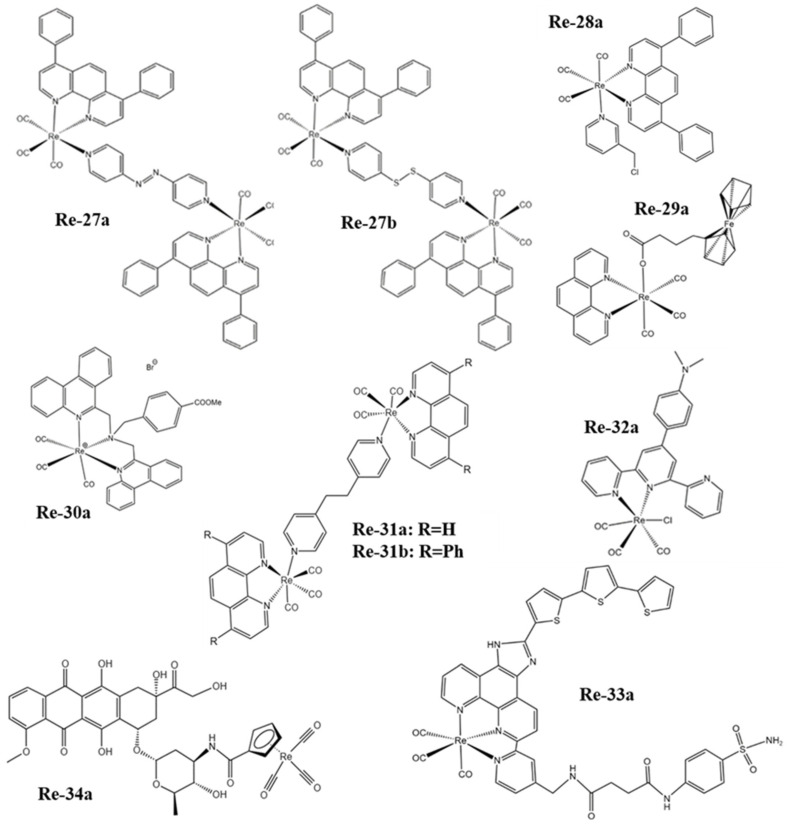
The structures of some Re complexes (Re-27a−Re-34a) involved in this review.

The atomic number of Re is 75 and its electron arrangement is [Xe]4f^14^5d^5^6s^2^. There are two natural isotopes ^185^Re (37.4%) and ^187^Re (62.6%) that exist in nature, which are potential candidates for medical applications [50]. However, ^188^Re is often used in cancer radiotherapy because of the relatively weak radiation emission of ^187^Re. Based on the fact that there are many valence electrons in the outer layer of rhenium, it has a variety of oxidation states, which makes it possible to form different geometries with many different ligands. Therefore, Re complexes have been widely studied in the field of anticancer for a long time. At present, the photoluminescence, lipophilicity, cytotoxicity, cell uptake, biological distribution, pharmacology, and toxicology of Re complexes can be regulated by changing their ligands [51]. The structural formulas of rhenium complexes mainly involved in this paper are shown in Figure 1, Figure 2 and Figure 3. Meanwhile, some inorganic nanomaterials of Re have strong absorption capacity in the near-infrared (NIR) region and a high Z property. Therefore, studies on multimodal imaging-guided synergistic cancer therapy have been popular in recent years.

In this review, we mainly summarized the different therapeutic methods and common imaging techniques of Re in the treatment of cancer. First of all, we introduced the general development of radionuclide ^188^Re in radiotherapy and the different substances commonly labeled with it, as well as some innovative applications in nuclear medicine in the past decade. Secondly, the mechanism of various Re complexes in cancer treatment was introduced in detail from the aspects of regulating the expression of related proteins, the photodynamic effect, interaction with DNA, destroying mitochondrial function, and so on. Then, some special Re nanomaterials were introduced, which mainly use photothermal action to ablate tumors. Finally, the Re-related imaging techniques were briefly described, and their advantages were reflected upon using different application examples. In a word, we focused on elucidating the mechanism of various Re compounds in cancer treatment, and introduced the corresponding imaging techniques, hoping to give some inspiration to researchers who develop new Re drugs for cancer diagnosis and treatment.

## 2. Study on Re in Cancer Therapy

### 2.1. Radioisotopes of Re for RT

^188^Re is a radionuclide that can be used in both imaging and therapy applications because it has 155 keV γ emission (for molecular imaging) and 2.12 MeV β emission (for RT), and the maximum tissue penetration range is 11 mm [52]. The physical half-life of ^188^Re is very short, only 16.9 h, which is very suitable for tumor therapy [53]. However, the physical half-life of ^186^Re is 90 h. Comparing the two of them, ^188^Re is more widely used. The initial study of the Re element in nuclear medicine is generally designed regarding the existence of ^99m^Tc. Researchers expect that Re radiopharmaceuticals will show a biological distribution pattern similar to Tc radiopharmaceuticals. Because the physical properties of drugs in nuclear medicine mainly depend on size, shape, and charge, similar Re radiopharmaceuticals and Tc radiopharmaceuticals cannot be distinguished by biological systems [54]. However, the chemical properties of similar Re and Tc radiopharmaceuticals are unpredictable. For example, the highly oxidized complexes of Re are more difficult to be reduced than similar Tc complexes [55]; so, these different chemical properties can provide the basis for biological differences between analogs, which is where researchers develop the dynamics of Re radiopharmaceuticals.

Some radiopharmaceuticals labeled with ^188^Re began to be used in the treatment of various diseases after the development and improvement of the ^188^W/^188^Re generator [56]. For example, Venkatesan et al. developed Re sulfide (^188^Re) colloidal drugs as radioactive synovial excision agents and potential drugs for tumor therapy, which not only have good stability and low manufacturing cost, but also prevent tumor radiation leakage by controlling the particle size [53,55,57]. Because ^188^Re is obtained in carrier-free form, it has very high specific activity, so it is feasible to radiolabel monoclonal antibodies without affecting immunoreactivity. There are some studies that have reported both direct labeling of the antibody itself [58,59] and indirect labeling of the chelating groups attached to the antibody [60]. ^188^Re can be labeled not only with monoclonal antibodies, but also with receptor-binding peptides, such as somatostatin analog (RC-160) [61,62], α-melanocyte-stimulating hormone (α-MSH) [63], etc. ^188^Re can also be used to label microspheres; the diameter of 1–5 μm is suitable for the treatment of rheumatoid arthritis and other cancers [64,65], while those larger than 10 μm can be used for liver cancer treated via hepatic artery administration [66,67].

In the past decade, the development of ^188^Re in nuclear medicine has been slow, with few innovative studies being reported [68,69,70]. However, the research on radioactive rhenium has not stopped. Wilber et al. [71] coupled ^188^Re with attenuated Listeria monocytogenes-binding antibodies, resulting in unique Listeria radioactivity for the treatment of pancreatic cancer, and the therapeutic effect was remarkable. This is the first report of using live attenuated bacteria to deliver high-level radioactive drugs to metastatic foci and specifically kill tumor cells in vivo. Aslan et al. [25] utilized the radioactive Re carbonyl complex to label the magnetic ferritin nano-cage for the first time, and realized the functions of magnetic targeted RT and MRI at the same time, and the Re carbonyl complex attached to magnetic ferritin had a good application prospect with a high labeling rate and low toxicity. Chao et al. [72] designed a self-sensitized and near-infrared enhanced radioisotope therapy nanomaterial (^188^Re-WS_2_-PEG) using the function that tungsten can absorb the ionizing radiation produced by ^188^Re, which cannot only greatly alleviate the hypoxia in the tumor, but also help to overcome the radiation resistance associated with hypoxia, and significantly improve the therapeutic effect. 

In recent years, there have also been some reviews on the production and application of Re radioisotopes in detail [73,74], and scholars who wish to further understand this field can consult this literature. Although there are still researchers actively investigating ^188^Re radiopharmaceuticals to demonstrate their potential clinical applications in treating a variety of benign and malignant diseases, overall enthusiasm for research is declining due to the high expected cost.

### 2.2. Re Complexes in Cancer Therapy

Since the development of Re chemistry, the coordination chemistry of Re has been a popular topic for study and one favored by many researchers. A large number of Re-based complexes have been synthesized, and the main application fields have involved catalysis, biomedicine, small molecule activation, photochemistry, new electronic materials, and so on [75]. Although there have been some reviews on the application of Re complexes in cancer treatment, most of the mechanisms of the cancer treatment have been unclear or even not discussed [70,75,76,77,78,79,80]. Thus, this review mainly focuses on elucidating the mechanism of Re complexes in cancer treatment, and their mechanisms of action on cancer cell lines are listed in Table 1.

#### 2.2.1. Regulating the Expression of Related Proteins Causes Cancer Cells to Die

It has been reported that Re complexes interact with histidine, glutamic acid, aspartic acid, and especially C-terminal carboxylic acid groups in these amino acids [115]. Therefore, some special Re complexes can affect the normal function of some proteins, thus further affecting cell activity. Because Re complexes interact with proteins, they have different effects on cells, which may disrupt the cell cycle and inhibit cell growth, and may also lead to apoptosis or necrosis.

Simpson et al. [81] prepared a Re complex Re-1a (Figure 1) and explored the anticancer properties of pancreatic cell lines. The results showed that the complex could not induce apoptosis or destroy the cell membrane of pancreatic cancer cells, but played a role as a cell inhibition drug. Further studies have found that the complex can inhibit the phosphorylation of Aurora-A kinase, while Aurora-A plays a key role in regulating the cell cycle and mitosis as well as many important carcinogenic signal pathways, resulting in a significant decrease in the number of cancer cells in the G1 phase, and most of the cancer cells are stagnated in the G2/M phase. The Re complex Re-2a (Figure 1) also mainly inhibits the growth of cancer cells rather than killing cancer cells in vivo, which mainly locates in lysosomes and induces cytoplasmic vacuolization [82].

**Figure 4:**
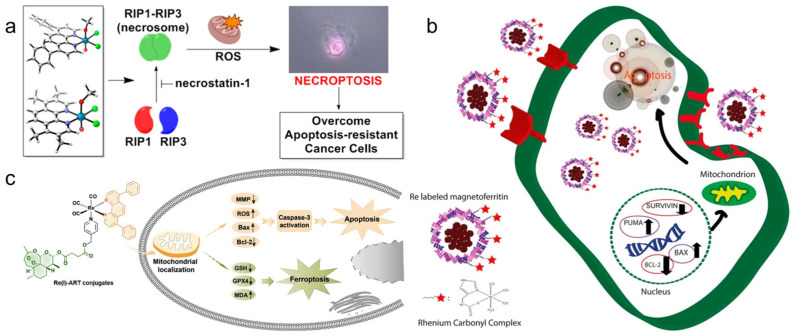
(**a**) Schematic diagram of the process of Re-4a and Re-4b inducing cell necrosis (reproduced with permission from Ref. [84], Copyright 2015, American Chemical Society); (**b**) schematic diagram of the process of Re-magnetoferritin nanoparticles inducing cell necrosis (reproduced with permission from Ref. [116], Copyright 2018, Elsevier B.V.); (**c**) schematic diagram of the process of Re-6a inducing apoptosis and ferroptosis of cancer cells (reproduced with permission from Ref. [86], Copyright 2021, Elsevier B.V.).

Konkankit et al. [83] synthesized eighty Re complexes containing diimine ligands via the microwave-assisted combination method, and found that the complex Re-3a (Figure 1) treated with 1% dimethyl sulfoxide (DMSO) at 10 μM could induce more than 95% cell death. To explore the mechanism of cell death induced by Re-3a, the results showed that Re-3a could not induce cell cycle arrest or phosphatidylserine inversion to the outer membrane, but could cause the rapid rupture of the plasma membrane. Morphological studies showed that most of the cells were round and cytoplasmic excretion was observed. All of these results proved that Re-3a causes cancer cell necrosis. Additionally, two kinds of Re complexes Re-4a and Re-4b (Figure 1) synthesized by Suntharalingam et al. [84] can also induce RIP1-RIP3-mediated necrosis rather than uncontrolled necrosis or apoptosis in A549 cells. The results showed that the overall expression levels of RIP1 and RIP3 in A549 cells remained unchanged with an increase in the dose of the complex. Therefore, the cell death induced by complexes depends on the formation of the RIP1-RIP3 complex, not on the expression level of single protein kinase. The complexes also lead to the production of reactive oxygen species (ROS) and the depletion of mitochondrial membrane potential (Figure 4a).

The first apoptosis signal (Fas) membrane protein is a receptor related to cell differentiation, proliferation, and apoptosis. The Fas receptor has some soluble subtypes and lacks a transmembrane domain. The Re complex Re-5a (Figure 1) can inhibit the soluble form of the Fas receptor so that the membrane Fas domain can be used to capture apoptosis signals. Meanwhile, the Fas-mediated exogenous apoptosis pathways can interfere with each other through cystatin, Bid cleavage, and endogenous pathways, resulting in the cleavage of Bcl family proteins and the activation of Bax proteins in mitochondria. Finally, it leads to an increase in pro-apoptotic Bax-α and induces cell death [85]. Other studies have shown that Re-magnetoferritin nanoparticles can enter adenocarcinoma cells through receptor-mediated endocytosis. Cancer cells show significantly higher uptake and cytotoxicity than normal cells. By analyzing the expression of related genes, the results showed that the pro-apoptotic Puma and Bax genes were significantly up-regulated, while the anti-apoptotic Bcl-2 and survivin genes were significantly down-regulated, resulting in apoptosis-induced cell death (Figure 4b). Additionally, if ^188^Re is used for Re-magnetoferritin, it can provide an attractive platform for local RT, because it not only has magnetic targeting ability but can also enhance the contrast in MRI signals [116].

Ye et al. [86] reported that a Re complex Re-6a (Figure 1) coupled with artesunate can induce apoptosis through mitochondrial membrane depolarization, adenosine triphosphate (ATP) consumption, and increased production of ROS and caspase-3. In addition, Re-6a can also inhibit the expression of glutathione peroxidase 4 (GPX4) and induce the accumulation of lipid peroxidation, resulting in cell ferroptosis (Figure 4c). Therefore, Re-6a can induce apoptosis and ferroptosis to inhibit cancer cell proliferation at the same time, so it can produce high cytotoxicity and enhance the treatment efficacy of cancer. The cluster Re complex Re-7a (Figure 1) with strong reducing ability can also produce a similar effect. As an active antioxidant, Re-7a can interfere with the glutathione system at the substrate level and enzyme level, and affect the regulation of intracellular ATP levels to cause cell apoptosis and death. Because it can also interact with phosphate groups, it can also participate in the process of ceramide-sphingosine-sphingosine-1-phosphate rheostat, which determines the balance between cell survival and apoptosis [87]. The cancer cells treated with the Re complex Re-8a (Figure 1) also showed the characteristics of apoptosis, such as cell contraction and nuclear fragmentation, but the specific process of regulation remains to be further studied [88].

King et al. [89] reported a Re complex Re-9a (Figure 1) that can induce endogenous apoptosis; so, it shows good anticancer activity in many cancer cell lines. The results show that the complex can trigger the accumulation of misfolded proteins, resulting in endoplasmic Re stress and an unfolded protein reaction. Moreover, the accumulation of misfolded proteins also leads to the phosphorylation of eIF2a, which starts autophagy and shuts down the whole protein translation; it also up-regulates apoptosis-promoting protein ATF4, which in turn leads to the expression of the apoptosis-promoting protein CHOP, thus inducing mitochondrial membrane depolarization and the release of cytochrome c. This process ultimately leads to the activation of caspase and the initiation of apoptosis.

#### 2.2.2. PDT Antiproliferation of Cancer Cells

Many Re complexes have good photochemical reactivity and a long triplet lifetime. Therefore, the cytotoxicity of these complexes will increase significantly after light irradiation. This is mainly due to the production of singlet oxygen (^1^O_2_) or other ROS in light stimulation. Therefore, after adjusting the lipophilicity of these complexes, they can be used as photosensitizers in PDT [79,117,118].

**Figure 5:**
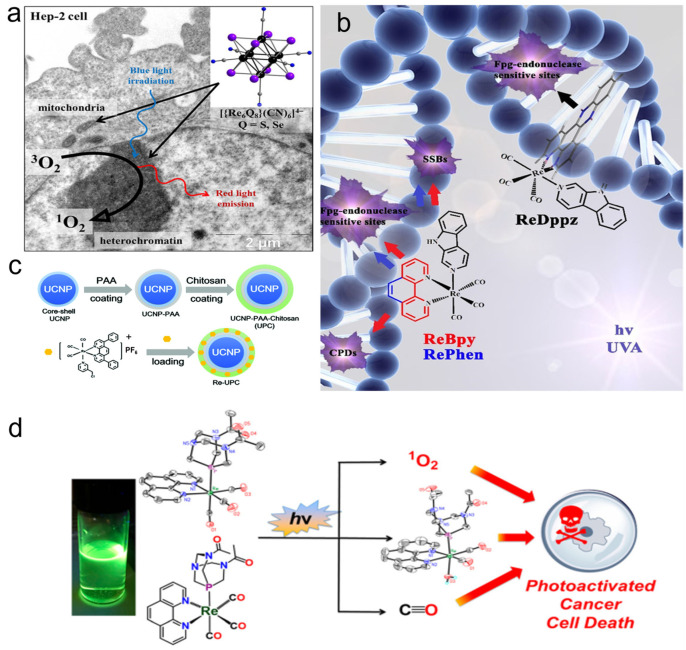
(**a**) Structure and working mechanism diagram of octahedral Re complex (reproduced with permission from Ref. [119], Copyright 2017, American Chemical Society); (**b**) main photochemical pathways involved in the DNA damage photosensitized by Re-complexes (reproduced with permission from Ref. [120], Copyright 2018, Wiley-VCH); (**c**) schematic representation for the synthesis of the Re-14a-loaded UCNPs (reproduced with permission from Ref. [94], Copyright 2003, Royal Society of Chemistry); (**d**) schematic diagram of photolysis of Re-15a (reproduced with permission from Ref. [95], Copyright 2018, American Chemical Society). UCNPs, upconversion nanoparticles.

Pan et al. [90] explored the therapeutic effect of two binuclear phosphorescence rhenium tricarbonyl complexes Re-10a and Re-10b (Figure 1) containing carboxyl derivatives as PDT agents. Studies on the mechanism of action showed that Re-10b could induce cancer cells to overproduce ROS, damage lysosomes, and induce apoptosis. Other studies have shown that the octahedral Re complex ([(Re_6_Q_8_)(CN)_6_]^4−^, Q = S, Se, Te) can also produce ^1^O_2_ when used in blue light. Among them, the selenium-contained complex has the best performance in absorption and the ^1^O_2_ generation yield (Figure 5a) [119].

Wähler et al. [91] have shown that the Re complex Re-11a (Figure 1) can induce apoptosis by producing ^1^O_2_ under light irradiation. Further studies have shown that the Re complex Re-11a could up-regulate the expression of caspases-3 and caspase-7 and increase the number of sub-G1 cells under green light irradiation. The condition of cells after treatment is consistent with that of NHIK3025 cancer cells treated with photosensitizer hematoporphyrin reported by Moan et al. [121]. Then, they reported Re(I) pyridine carbazole complexes with light-induced antiproliferative activity, which could not only produce ^1^O_2_ under red light, but also inhibit the expression of cancer-related protein kinases [122].

Three kinds of Re complexes (called ReBpy, RePhen, and ReDppz, as seen in Figure 5b) were reported by Maisuls et al. [120]. These complexes can produce ROS and further damage DNA under light excitation. The types of DNA damage induced by the three Re complexes depend on the chemical properties of ligands and the type and extent of ROS produced after photoexcitation. ^1^O_2_ is the key oxidant to induce purine oxidation. The results showed that both ReBpy and RePhen induced DNA single-strand breaks and formamidopyrimidine-DNA-glycosylase sensitive base modification, while ReDppz only induced the latter type of modification. This is because the former two interact with DNA mainly through electrostatic interaction and groove binding, while the main interaction mode of the latter is insertion into stacked bases. The Re complex Re-12a (Figure 1) with a porphyrin structure has a similar function; it cannot only produce ^1^O_2_, but also the porphyrin structure can be embedded into plasmid DNA and bind to it, inducing satisfactory PDT performance [92].

Leonidova et al. [93] studied two Re(I) tricarbonyl complexes, Re-13a and Re-13b derivatives (Figure 2). Because Re-13a and Re-13b have been proven to be excellent ^1^O_2_ generators in a lipophilic environment, the ^1^O_2_ generation yield is about 75%. Therefore, to improve their selectivity, they were combined with two types of peptides (a nuclear localization signal and a derivative of the neuropeptide bombesin). The results showed that this significantly enhanced its accumulation in the nucleolus, and the cytotoxicity increased significantly after light irradiation. This is because singlet oxygen and a small amount of superoxide caused damage to DNA.

Hu et al. [94] fabricated the photoactivable Re(I) complex Re-14a (Figure 2) with lanthanide-doped upconversion nanoparticles (UCNPs). The Re-14a can generate ROS and release carbon monoxide (CO) by absorbing the ultraviolet (UV) light generated by UCNPs under 980 nm light irradiation, which enhances the damage to cells (Figure 5c). Marker et al. [95] found that the Re(I) tricarbonyl complex Re-15a (Figure 2) containing water-soluble phosphine can also release the Re dicarbonyl complex and CO and produce ^1^O_2_ during photolysis in water (Figure 5d). Cytotoxicity experiments showed that ^1^O_2_ sensitization played a role in the mechanism of action of the compound. However, Re dicarbonyl light-irradiation products and CO also caused certain phototoxicity.

#### 2.2.3. Interaction with DNA to Inhibit Cancer Cells

The toxicity of the interaction of the Re complex with DNA to cancer cells has been well demonstrated. Depending on the type of ligand, the way it interacts with DNA will be different, which will be explained in detail below.

Wilder et al. [123] synthesized a series of Re complexes XRe(CO)_3_Z (X = 2-pyridin-2-ylpyridine, 1,10-phenanthroline, 5-methyl-1,10-phenanthroline, 2,9-dimethyl-1,10-phenanthroline, 5,6-dimethyl-1,10-phenanthroline, 4,7-diphenyl-1,10-phenanthroline, 2,9-dimethyl-4,7-diphenyl-1,10-phenanthroline, 4,7-dimethyl-1,10-phenanthroline, 3,4,7,8-tetramethyl-1,10-phenanthroline and Z = p-toluenesulfonate, 1-naphthalenesulfonate, 2-naphthalenesulfonate, picolinate, nicotinate, aspirinate, naproxenate, flufenamate, ibuprofenate, mefenamate, tolfenamate, and N-acetyl-tryptophanate) and studied their biological properties. The studies show that they interact with DNA via partial insertion, and the anticancer activity is the same as the DNA-binding activity, which increases with the lipophilicity of the complexes. The Re atoms of the Re complexes Re-16a [96] and Re-17a [97] (Figure 2) coordinate with three planar carbonyl and planar polypyridine aromatic ligands to form an octahedral structure. Because of the planarity of aromatic phenanthroline rings, the complexes are forced to insert into the DNA bases. The studies show that the Re complex Re-17a does not bind to DNA through the covalent interaction of nitrogen bases (adenine, cytosine, guanine, and thymine); so, the two Re complexes bind to DNA via partial insertion.

Zobi et al. [98] studied the reaction of the Re complex Re-18a (Figure 2) with purine and adenosine. They found that the two bases of purine can coordinate with the center of the Re atom through the N7 atom, resulting in a fairly stable complex with a slow on/off rate, and the on/off rate and activity are similar to those of cisplatin. These results suggest that the cytotoxic mechanism of this Re complex may be similar to that of cisplatin. Later, they studied the Re complex Re-19a (Figure 2) [99] and considered that the interaction between the Re atom in Re-19a and DNA may involve the phosphate skeleton. However, the four-membered ring produced by binding to the phosphate skeleton is thermodynamically unfavorable, while the interaction with the nitrogen base is much easier. Therefore, the Re atoms are most likely to bind to two DNA bases, that is, selectively interact with the adjacent guanines in DNA, resulting in structural changes in DNA. Additionally, in their subsequent study, it was further found that the two N7 coordinated guanines partially exist in head-to-head orientation [124].

The Re complexes Re-20a [100] and Re-21a [101] (Figure 2) both interact with DNA through groove binding, and Re-20a has concentration-dependent DNA cleavage activity due to its potential to produce ROS that damage DNA. In addition, the interaction between N and DNA will maintain its overall structure, while the non-coordination ligand benzene dione interacts via embedding in DNA. The structures of the Re complexes Re-22a [102] and Re-23a [103] (Figure 2) are also in accordance with the standard of small groove-binding DNA. The quinoline ring will selectively bind to the Amit site of DNA, and the positively charged Re chelate may interact with the negatively charged DNA skeleton. However, this interaction and embedding do not result in topoisomerase inhibition.

#### 2.2.4. Destroy the Function of Mitochondria and Kill Cancer Cells

Studies have shown that mitochondria are related to a variety of signal pathways that control apoptosis, so the design of drugs targeting mitochondria for cancer treatment has been attracting the attention of researchers. These drugs generally treat cancer by changing the permeability of the mitochondrial membrane, reversing mitochondrial metabolism, or increasing the production of ROS. 

**Figure 6:**
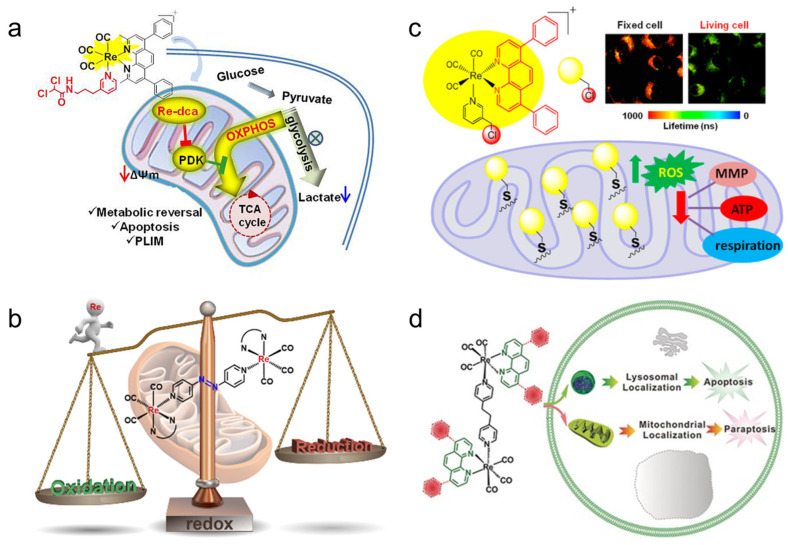
(**a**) Structure and action mechanism diagram of Re-24a (reproduced with permission from Ref. [104], Copyright 2018, Elsevier B.V.); (**b**) structure and action mechanism diagram of Re-27a (reproduced with permission from Ref. [107], Copyright 2019, American Chemical Society); (**c**) structure and action mechanism diagram of Re-28a (reproduced with permission from Ref. [108], Copyright 2017, American Chemical Society); (**d**) structure and action mechanism diagram of Re-31a and Re-31b (reproduced with permission from Ref. [111], Copyright 2016, Wiley-VCH).

The Re complex Re-24a (Figure 2) reported by Yang et al. [104] can selectively enter cancer cells and be located in mitochondria, eventually leading to caspase-dependent apoptosis by inducing mitochondrial dysfunction and metabolic disorder. The results proved that Re-24a can inhibit the activity of pyruvate dehydrogenase kinase, thus realizing the metabolic reversal from glycolysis to glucose oxidation (Figure 6a). In addition, Re-24a can significantly inhibit the metastasis and invasion of cancer cells and has good anti-angiogenic activity in zebrafish embryos. The Re complex Re-25a (Figure 2) can also inhibit cell migration, invasion, and angiogenesis by down-regulating the expression of MMP-2 and VEGF. Moreover, Re-25a can also up-regulate the expression level of cytochrome C and PARP, increasing ROS production and a decrease in ATP synthesis. In addition, it can up-regulate the expression of Bax and down-regulate the expression of Bcl-2, and induce apoptosis through the caspase pathway [105]. However, although the Re complex Re-26a (Figure 2) also targets mitochondrial respiration, resulting in reduced cell metabolism, it will increase glycolysis. In addition, the effect of mitochondrial activity mainly involves two modes. At lower concentrations, it can increase respiration by increasing proton transport through the mitochondrial inner membrane, while at higher concentrations, it can effectively block respiration. The results showed that the complex could induce the production of ROS in cells and finally lead to apoptosis [106].

Wang et al. [107] reported that the Re complexes Re-27a and Re-27b (Figure 3) can also target mitochondria and cause oxidative stress, thus leading to mitochondrial dysfunction, which not only reduces cell metabolism but also interferes with glutathione metabolism, ultimately inducing cancer cell necrosis and caspase-dependent apoptosis (Figure 6b). The Re complex Re-28a (Figure 3) can also cause caspase-dependent apoptosis of cells, and its chloromethyl pyridyl moiety is partially fixed in mitochondria by nucleophilic substitution with the mercaptan group of proteins. A mechanism study showed that Re-28a can cause mitochondrial damage, decrease its membrane potential, and inhibit respiration, thus leading to less ATP and more ROS (Figure 6c) [108].

The Re complex Re-29a (Figure 3) synthesized by Skiba et al. [109] selectively accumulates in mitochondria through a special dissociation driving mechanism. Firstly, ligands with ester groups dissociate in cells to produce fac-[Re(CO)_3_(1,10-phenanthroline)]^+^. Then, the secondary products produced by the rapid reaction of these cations with various intracellular molecules are absorbed by negatively charged mitochondrial membranes and covalently bind to mitochondria. Finally, it increases the production of ROS and induces cell phototoxicity. The Re complex Re-30a (Figure 3) also has an ester structure and thus similarly accumulates in mitochondria, and can activate both the receptor initiation pathway and the mitochondrial-dependent pathway to induce apoptosis. The receptor initiation pathway partly depends on the activation of caspase-8 and caspase-3, while the signal pathway participated in by mitochondria is usually related to the release of cytochrome c, which usually leads to a decrease in mitochondrial membrane potential. In addition, Re-30a can overcome drug resistance due to an overexpression of p-glycoprotein; so, it may induce apoptosis even in drug-resistant cell lines [110].

Re complexes Re-31a and Re-31b (Figure 3) [111] can affect the p53 signal pathway. The activation of the p53 pathway plays an important role in apoptosis and paraptosis. Paraptosis is a type of non-apoptotic cell death, which is characterized by cytoplasmic vacuolization and nuclear integrity, and sometimes mitochondrial swelling [125,126]. The results show that Re-31a can affect endocytosis because it is mainly located in the lysosome, which is an important part of endocytosis. In addition, Re-31a induces caspase-independent apoptosis, and the production of ROS is not involved in this apoptosis. Re-31b can affect the “tricarboxylic acid cycle” in the mitochondrial matrix, and ROS plays an important role in Re-31b-induced paraptosis. So, Re-31b exerts anticancer activity by affecting mitochondrial function (Figure 6d).

#### 2.2.5. Other Ways to Kill Cancer Cells

In the field of cancer treatment, the Re complex not only has several common treatment methods described above but also many researchers have recently made innovations based on previous research to develop new cancer treatment methods [127]. These methods have good development prospects and it is hoped that they will point out some directions for the development of the novel Re complex as a cancer therapeutic drug.

**Figure 7:**
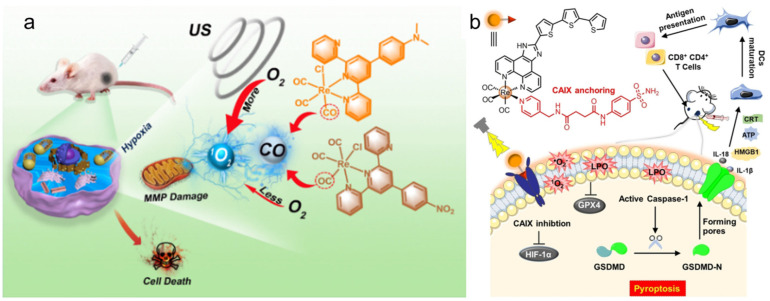
(**a**) The mechanism of the combination of SDT and CO gas therapy based on Re-32a (reproduced with permission from Ref. [112], Copyright 2023, Elsevier B.V.); (**b**) structure of Re-33a capable of inducing and self-reporting membrane rupture upon irradiation, as well as evoking pyroptosis and anti-tumor immunity (reproduced with permission from Ref. [113], Copyright 2021, Wiley-VCH).

The Re complex Re-32a (Figure 3) reported by Li et al. [112] can be used as a new type of SDT gas therapy for cancer, which can overcome the shortcomings of hypoxia and insufficient penetration depth in traditional PDT. Because Re-32a triggered by ultrasound can release CO in situ, higher levels of CO have pro-apoptotic and anti-proliferative activity on cancer cells [128,129], and the treatment of cancer based on CO is oxygen-independent. In addition, ultrasound stimulation can also produce ^1^O_2_. The results have shown that Re-32a can target mitochondria, damage mitochondria through CO and ^1^O_2_ to depolarize the mitochondrial membrane, and eventually lead to cancer cell death (Figure 7a).

The Re complex Re-33a (Figure 3) designed by Su et al. [113] has a carbonic anhydrase IX structure, which enables it to be immobilized on the cell membrane, and this structure can alleviate tumor hypoxia to a certain extent, thus contributing to the in situ production of ROS and improving PDT efficiency. Under the excitation of light, Re-33a can produce a large amount of ROS to make the cell membrane lipid peroxidation, which will increase the consumption of GPX4, and eventually cause gasdermin D-mediated cell scorch. Subsequently, a series of inflammatory cytokines and damage-related molecules are released at the treatment site, which stimulates dendritic cells to mature for antigen presentation and ultimately activates an adaptive immune response in vivo that destroys both the primary tumor and the growth of the distal tumor (Figure 7b).

### 2.3. Re Nanomaterials in Cancer Therapy

Nanomaterials usually have some unique physical and chemical properties different from the corresponding bulk materials; so, they have been widely studied in various fields and have been rapidly developed [130]. Some of these nanomaterials with large specific surface areas and unique optical and thermal properties have been gradually applied to the biomedical field. Some inorganic nanomaterials of rhenium also have these properties, thus inducing the attention of researchers. At present, the reported Re inorganic nanomaterials mainly include Re elemental nanomaterials, Re disulfide nanomaterials, Re trioxide nanomaterials, etc. Due to the strong near-infrared absorption capacity of these nanomaterials, photothermal treatment could be realized in cancer treatment, and sometimes other therapeutic methods are also used together. The biomedical application of Re-based nanomaterials is summarized in Table 2.

**Figure 8:**
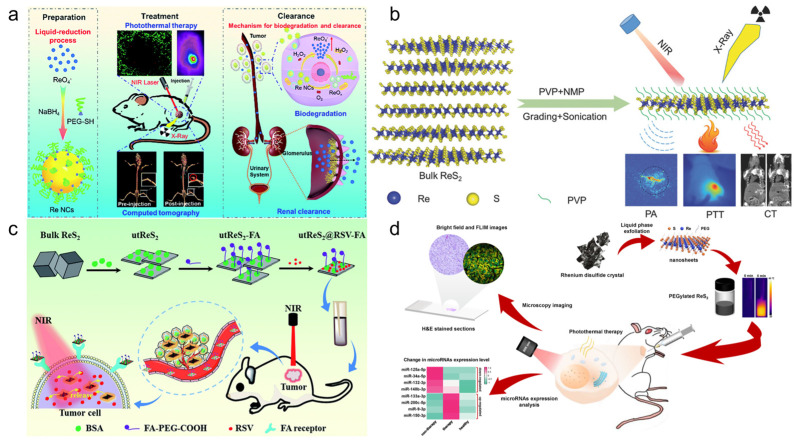
(**a**) Schematic of degradable and renal-clearable Re NCs for tumor diagnosis and therapy (reproduced with permission from Ref. [131], Copyright 2010, Royal Society of Chemistry); (**b**) scheme of the exfoliation process of PVP capped ReS_2_ nanosheets for bimodality PA and CT-imaging-guided photothermal therapy (reproduced with permission from Ref. [132], Copyright 2018, Wiley-VCH); (**c**) a schematic illustration of the utReS_2_@RSV–FA synthesis for tumor-targeted chemo-photothermal therapy (reproduced with permission from Ref. [135], Copyright 2011, Royal Society of Chemistry); (**d**) schematic illustration of PEG−ReS_2_ nanosheets employed for in vivo breast cancer therapy study (reproduced with permission from Ref. [134], Copyright 2022, MDPI). NCs, nanoclusters; PVP, polyvinyl pyrrolidone; CT, computed tomography; PA, photoacoustic imaging; PEG, polyethylene glycol.

Miao et al. [131] reported a simple liquid reduction method for the synthesis of polyethylene glycol Re nanoclusters (NCs). Re NCs have a high photothermal conversion efficiency (33.0%) and can effectively ablate tumors (100%). They are sensitive to H_2_O_2_ and can be degraded into biocompatible ReO_4_^−^ for renal clearance (Figure 8a). In addition, they also reported a method for the synthesis of colloidal ReS_2_ nanosheets with ultrasound-probe-assisted liquid peeling, which can be fully dispersed in a physiological environment after polyvinyl pyrrolidone (PVP) modification and can effectively ablate tumors with a photothermal conversion efficiency of 79.2% (Figure 8b) [132].

Shen et al. [133] synthesized a ReS_2_ nanosheet with uniform size, which can stably exist in various physiological solutions after being modified with polyethylene glycol (PEG). The results show that ReS_2_-PEG has strong near-infrared light and X-ray absorption capacity, so it can be used not only as a photothermal agent for PTT, but also as a sensitizer for RT. In addition, the results of the photothermal agent combined with RT showed that the photothermal agent could significantly improve the hypoxic environment of tumors, thus reducing the RT resistance of tumors and finally achieving significant therapeutic effects. Song et al. [134] also synthesized PEG-ReS_2_ nanosheets with different methods, which also have ideal photothermal conversion efficiency and could effectively ablate tumors. In addition, the micro-RNA (miRNA) expression analysis and evaluation methods also showed that PEG-ReS_2_ nanotablets had good therapeutic effects (Figure 8d).

Huang et al. [135] prepared ultra-thin Re disulfide nanosheets using the bovine-serum-albumin-assisted ultrasonic peeling method, further loaded resveratrol and folic acid, and finally formed a nanocomposite with good biocompatibility and high near-infrared absorbance (called utReS_2_@RSV–FA). This composite not only has significant tumor targeting ability, but also shows good photothermal effect under 808 nm laser irradiation. Therefore, it is possible to realize a dual response of pH/temperature to deliver drugs, and achieve a combination of chemical–photothermal therapy (Figure 8c). Wang et al. [136] synthesized ReS_2_ NPs via the one-pot method under mild conditions. The size of ReS_2_ NPs was less than 10 nm, and they had good monodispersity and water solubility and strong near-infrared absorption ability. The results showed that ReS_2_ NPs also have good biocompatibility and low cytotoxicity, which can be used for PTT.

The aqueous dispersion of ReO_3_ NCs synthesized by Zhang et al. [137] has high surface plasmon resonance absorbance in the near-infrared region, and its photothermal conversion efficiency is as high as 57.0%, which can also be used for PTT of cancer. ReO_3_ NCs not only have tumor-targeting ability, but also show pH-dependent oxidative degradation. They exist stably in the weakly acidic tumor microenvironment but can be degraded effectively in a normal physiological environment, to realize efficient and safe tumor therapy.

## 3. Application of Re in Biological Imaging

Many Re-related compounds have potential in biological imaging. Most Re complexes can emit phosphorescence, so they are suitable for bio-optical imaging. Optical imaging is widely used from the cell to the in vivo level, but it is limited by the wavelength of excitation. The limited depth of light penetration at the in vivo level makes the imaging resolution low. Based on the high atomic number characteristics of Re, Re complexes or nanomaterials can also be used as CT sensitizers to increase the imaging signal-to-noise ratio. Thus, rhenium compounds have biomedical imaging ability for the substances.

### 3.1. Optical Imaging

Re(I) tricarbonyl complexes are widely used in biological imaging because of their unique luminescent characteristics, such as long luminescent lifetime, large Stokes shift, high quantum yield, and strong absorption in the infrared transparent window (1800–2200 cm^−1^) of biological media [117,138,139,140,141,142].

**Figure 9:**
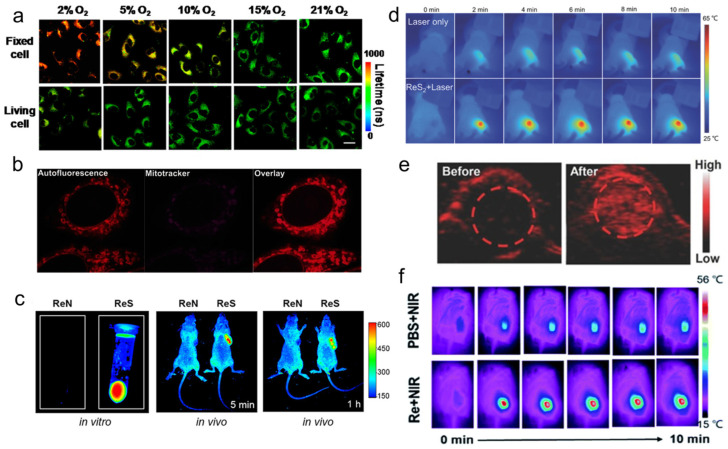
(**a**) PLIM images of Re-14a-treated fixed and living A549 cells under different oxygen partial pressures at 37 °C (reproduced with permission from Ref. [108], Copyright 2017, American Chemical Society); (**b**) magnified images showing an overlay of Cp-Dox autofluorescence and MitoTracker fluorescence in HeLa cells, suggesting an accumulation of the Dox conjugate in the mitochondrial membrane (reproduced with permission from Ref. [114], Copyright 2016, Wiley-VCH); (**c**) fluorescence emission of Re-27a and Re-27b in vitro and in vivo (reproduced with permission from Ref. [107], Copyright 2019, American Chemical Society); (**d**) thermal images of PVP capped ReS_2_ in in vivo PTT (reproduced with permission from Ref. [132], Copyright 2018, Wiley-VCH); (**e**) PA images of tumors in mice before and 24 h after i.v. injection of ReS_2_-PEG (reproduced with permission from Ref. [133], Copyright 2017, Wiley-VCH); (**f**) thermal imaging of tumor-bearing mice after injection of PBS or Re NCs (reproduced with permission from Ref. [131], Copyright 2010, Royal Society of Chemistry). PLIM, phosphorescence lifetime imaging; PVP, polyvinyl pyrrolidone; PTT, photothermal therapy; PA, photoacoustic; PEG, polyethylene glycol; PBS, phosphate buffer saline; NCs, nanoclusters.

Yang et al. [108] found that the phosphorescence intensity and lifetime of the Re-14a complex (Figure 1) showed excellent oxygen sensitivity. Specifically, the higher the O_2_ concentration, the weaker the phosphorescence intensity and the shorter the lifetime. Therefore, they monitored the O_2_ consumption in cancer cells using phosphorescence lifetime imaging, and further studied the changes in mitochondrial metabolism (Figure 9a). In addition, they found that the Re complex Re-24a (Figure 2) also had the same properties in later research [104]. 

Imstepf et al. [114] observed that Re-34a (Figure 3) could selectively stain the mitochondrial membrane using a fluorescence microscope in the study of transferring doxorubicin to mitochondria via the rhenium complex, which indicated that Re-34a interacted with the membrane through lipophilicity, and finally proved that the Re complex could relocate doxorubicin from the nucleus to mitochondria (Figure 9b). Wang et al. [107] studied the optical imaging performance of intratumoral injection of Re-27a and Re-27b (Figure 2), and the results showed that Re-27b had better emission ability in vivo (Figure 9c).

Because infrared imaging does not involve electronic transition, photobleaching does not occur, but the sensitivity is usually not satisfactory. Most Re inorganic nano-materials used in tumor therapy have made use of their excellent photothermal conversion ability, so that they have the function of infrared thermal imaging, and usually have the ability of PA imaging. In the process of tumor treatment, infrared thermal imaging, PA, and CT imaging are usually combined to help guide cancer treatment more accurately [132,133,137]. Miao et al. [132] and Shen et al. [133] have studied the infrared thermal imaging (Figure 9d) and PA imaging (Figure 9e) properties of different ReS_2_ nanomaterials. In addition, Miao et al. [131] later studied the infrared thermal imaging performance of PEGylated Re NCs (Figure 9f). These results show that Re compounds have better optical imaging ability.

### 3.2. CT Imaging

**Figure 10:**
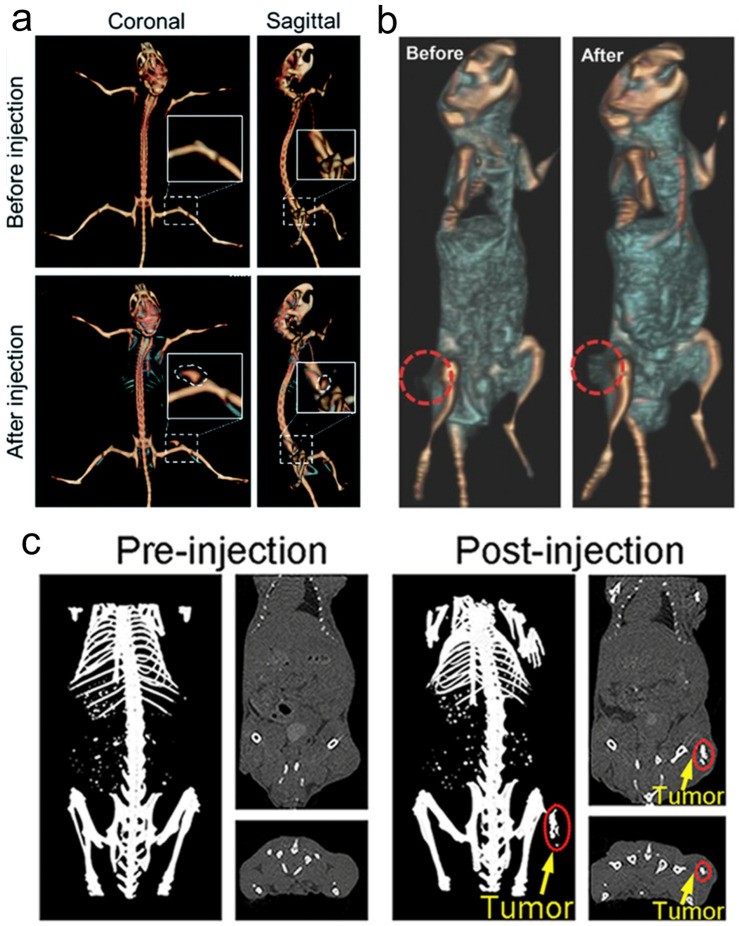
(**a**) CT imaging in vivo of 4T1 tumor-bearing BALB/c mice before and after injection of Re NCs (reproduced with permission from Ref. [131], Copyright 2010, Royal Society of Chemistry); (**b**) CT images of mice before and 24 h after i.v. injection of ReS_2_-PEG (reproduced with permission from Ref. [133], Copyright 2017, Wiley-VCH); (**c**) in vivo CT imaging before (pre) and after (post) i.t. injection with ReO_3_ NCs (reproduced with permission from Ref. [137], Copyright 2018, Elsevier B.V.). CT, computed tomography; PEG, polyethylene glycol; NCs, nanoclusters.

Because of the high atomic number property of Re itself (Z = 75), Re compounds usually have strong X-ray attenuation ability and can be used as CT contrast agents to enhance CT signals (Figure 10), which has been proven by many research results [131,133,137].

## 4. Conclusions

Re, with its unique physical and chemical properties, is favored by researchers in the diagnosis and treatment of cancer and has indeed contributed considerable achievements. Various ^188^Re-labeled radiopharmaceuticals are being used for different types of cancer treatment, and new radiopharmaceuticals are being developed. However, the high expected investment and inherent drawbacks of radiotherapy limit its development. By changing the ligand of Re complexes, various structures and functions of Re complexes could achieve different ways of killing cancer cells, and some of them could overcome tumor resistance or improve radiation resistance. However, the systematic design of and research into rhenium complexes as anticancer agents have been hampered by the fact that most studies have only been conducted at the cellular level and the mechanism by which the complexes kill tumors has not been thoroughly investigated. In recent years, some inorganic nanomaterials of Re have gradually attracted the attention of researchers, and a multifunctional therapeutic nanoplatform of multimodal imaging-guided cancer synergistic therapy has been developed. Because of the simple synthesis method of nanomaterials, they can be combined with other materials to improve their properties or expand their functions, which gives them a broad development prospect. The cancer diagnosis and treatment techniques and their characteristics mentioned in this review are shown in Table 3. 

Therefore, the design of Re compounds could consider combining radioisotopes with materials that can improve the anoxic microenvironment of tumors to alleviate radiation resistance, or could consider combining a variety of ligands that can cascade and influence the reaction within the tumor and increase selective uptake to improve the killing efficiency of cancer cells. We can also develop a new type of porous Re inorganic nanomaterial with a large specific surface area, and use it to load chemotherapy drugs or specific tumor-targeting drugs, or to construct heterojunctions with other semiconductor materials to achieve more efficient tumor therapy. We hope that this review can provide useful information for further research and the application of rhenium in biomedicine.

## Figures and Tables

**Table 1:** Mechanism of action of Re complexes and cancer cell lines they act on.

Compound	Cell Line	Mechanism of Action	Ref.
Re-1a	HPAF-II, ASPC1, CFPAC	Inhibit Growth	[81]
Re-2a	A2780, A2780CP70	Inhibit Growth	[82]
Re-3a	A2780, A2780CP70	Cell Necrosis	[83]
Re-4a	A549	Cell Necrosis	[84]
Re-4b	A549	Cell Necrosis	[84]
Re-5a	A431, DLD-1, A2780	Cell Apoptosis	[85]
Re-6a	HeLa	Cell Apoptosis and Ferroptosis	[86]
Re-7a	Guerink (T-8)	Cell Apoptosis	[87]
Re-8a	A549, HCT-15, HeLa, K562	Cell Apoptosis	[88]
Re-9a	A2780	Cell Apoptosis	[89]
Re-10a	A549	Photodynamic Therapy	[90]
Re-10b	A549	Photodynamic Therapy	[90]
Re-11a	HeLa	Photodynamic Therapy	[91]
Re-12a	HeLa, H460M2, HBL-100	Photodynamic Therapy	[92]
Re-13a	HeLa	Photodynamic Therapy	[93]
Re-13b	HeLa	Photodynamic Therapy	[93]
Re-14a	A2780, A2780cis	Photodynamic Therapy	[94]
Re-15a	HeLa, A2780, A2780CP70	Photodynamic Therapy	[95]
Re-16a	PC-3, MDA-MB-231, CCl-227	Insert DNA	[96]
Re-17a	PC-3	Insert DNA	[97]
Re-18a	/	Base Bind	[98]
Re-19a	/	Base Bind	[99]
Re-20a	/	DNA Groove Bind	[100]
Re-21a	T98G, PC3, MCF-7	DNA Groove Bind	[101]
Re-22a	A2780/AD	DNA Groove Bind	[102]
Re-23a	BeWo	DNA Groove Bind	[103]
Re-24a	NCI-1229	Cell Apoptosis	[104]
Re-25a	HepG2, HeLa, MCF-7, A549	Cell Apoptosis	[105]
Re-26a	MCF-7	Cell Apoptosis	[106]
Re-27a	HeLa, A549, MCF-7	Cell Apoptosis	[107]
Re-27b	HeLa, A549, MCF-7	Cell Apoptosis	[107]
Re-28a	A549	Cell Apoptosis	[108]
Re-29a	HeLa	Cell Apoptosis	[109]
Re-30a	NALM-6, BJAB, MelHO	Cell Apoptosis	[110]
Re-31a	HeLa, A549, HepG2	Cell Apoptosis	[111]
Re-31b	HeLa, A549, HepG2	Cell Paraptosis	[111]
Re-32a	4T1	Sonodynamic–Gas Therapy	[112]
Re-33a	MDA-MB-231	Photodynamic and Immunotherapy	[113]
Re-34a	HeLa	Cell Apoptosis	[114]

**Table 2:** Therapy methods of rhenium inorganic nanomaterials and cancer cell lines they act on.

Nanomaterials	Cell Line	Therapy Methods	Ref.
Re NCs	4T1	Photothermal Therapy	[131]
PVP capped ReS_2_	HeLa	Photothermal Therapy	[132]
ReS_2_-PEG	4T1	Photothermal Radiotherapy	[133]
PEG-ReS_2_	4T1	Photothermal Therapy	[134]
utReS_2_@RSV–FA	HepG2	Chemo-Photothermal Therapy	[135]
ReS_2_ NPs	4T1	Photothermal Therapy	[136]
ReO_3_ NCs	HeLa	Photothermal Therapy	[137]

**Table 3:** The cancer diagnosis and treatment techniques in this review.

Types of Materials	Therapy Methods	Advantage	Limitation
Re Radiopharmaceuticals	Radiotherapy	Simplicity of Operator	Kill Normal Cells
Re Complexes	Regulating the Expression of Proteins	Extensive Mechanism	Unclear mechanism
	Photodynamic Therapy	Minimally Invasive	Limited Penetration
	Interaction with DNA	Strong Targeting	Develop Resistance
	Destroy the Function of Mitochondria	High Selectivity	Low Intake
	Sonodynamic Therapy	Good Penetration	Oxygen Dependence
	Immunotherapy	Wide Effect	Slow Action
Re Nanomaterials	Photothermal Therapy	High Efficiency	Limited Penetration
**Types of Materials**	**Imaging Techniques**	**Advantage**	**Limitation**
Re Nanomaterials	Infrared Thermal Imaging	Non-photobleach	Temperature-dependent
Re Nanomaterials	Photoacoustic Imaging	High-resolution	Limited Penetration Depth
Re Complexes	Fluorescence Imaging	Sensitive	Limited Penetration Depth
Re Complexes	Phosphorescence Lifetime Imaging	Sensitive	Limited Penetration Depth
Re Nanomaterials	Computed Tomography	High-resolution	Tissue Damage

## Data Availability

Not applicable.

## Sample Availability

Samples of the compounds are not available from the authors.
